# Searching for Multimode Resonator Topologies with Adaptive Differential Evolution

**DOI:** 10.3390/s25206447

**Published:** 2025-10-18

**Authors:** Vladimir Stanovov, Sergey Khodenkov, Ivan Rozhnov, Lev Kazakovtsev

**Affiliations:** 1 Institute of Informatics and Telecommunications, Reshetnev Siberian State University of Science and Technology, Krasnoyarsk 660037, Russia; hsa1982sibsau@mail.ru (S.K.); levk@bk.ru (L.K.); 2 Institute of Business Process Management, Siberian Federal University, Krasnoyarsk 660041, Russia; ris2005@mail.ru

**Keywords:** multimode resonator, amplitude–frequency characteristics, microwave sensor, optimization, differential evolution

## Abstract

Microwave devices based on microstrip resonators are widely used today in communication, radar, and navigation systems. The requirements to these devices may include specific frequency-selective properties, as well as size and production costs. The design of resonators and filters are mostly performed manually, as the process requires expert knowledge and computationally expensive modeling, so practitioners are usually limited to tuning a chosen example from a set of known, typical topologies. However, the set of possible topologies remains unexplored and may contain specific constructions, which have not been discovered yet. In this study we propose an approach to automatically search the space multimode resonator topologies using a zero-order optimization algorithm and numerous computational experiments. In particular, a family of symmetrical resonators constructed out of four rectangles is considered, and the parameters are tuned by the recently proposed L-SRTDE algorithm. We state the problem of building the topology of a microwave device conductor with specified frequency-selective characteristics as an optimization problem, and the minimized function (target function) in this problem is based on the evaluation of the deviation between the specified frequency-selective characteristics and their values obtained via electrodynamic modeling. The experiments with two target function formulations have shown that the proposed approach allows finding novel topologies and automatically tune them according to the required frequency-selective properties. It is shown that some of the topologies are different from the known ones but still demonstrate high-quality properties.

## 1. Introduction

Microstrip resonators represent an important class of devices, which can be used in various applications, including telecommunication technologies [1], radar and navigation systems [2], measuring electrical characteristics of various materials [3,4], as well as non-invasive medical measurements [5,6]. The low cost and manufacturability allow them to be used to create bandpass filters, high- and low-pass filters, as well as diplexers and other devices [7,8]. Due to their small size, several millimeters, such resonators can be easily integrated with systems which require wireless communication, such as unmanned vehicles [9,10]. In some cases, such as radars and antennas, a wide range of frequencies should be covered, while in others, such as sensors, a very narrow bandwidth is required. The frequency-selective properties are tuned by bringing together several lowest oscillation modes while miniaturizing the scales of the construction [11].

The design of all mentioned microstrip resonators and more complex systems based on them is typically performed with a simulation-aided approach. The structure of the resonator is usually composed of a set of rectangles made of conductive material [7,12], which are placed on a substrate with a high dielectric constant. The amplitude-frequency characteristics (AFCs) of such devices is then modeled by simulation software with numeric electrodynamic analysis. This step requires a large amount of calculations; however, with modern hardware capabilities, it is possible to run multiple simulations in order to tune the structure for the specific application [13]. The widely known optimization techniques are often a part of the popular software products for the design of microwave devices. These include Nelder Mead algorithm, Trust Region Framework, Genetic Algorithm, Particle Swarm Optimization, and some other approaches. These tools reduce the amount of manual work required to design a specific device for the application in hand; however, they are limited to tuning a known structure, but are incapable of creating new ones.

Several research studies focused on designing microwave filters and other devices automatically; for example, in [14], the deep reinforcement learning (DRL) approach was proposed, and a relational induction neural network (RINN) was proposed. This network is capable of determining the relationship between structure parameters without prior knowledge. The authors have shown that their approach is capable of designing filters of up to the sixth order. Some other studies used genetic algorithms or binary particle swarm optimization with a pixelization strategy to design ultracompact planar filters [15] or near-field sensors [16]. In [17], the authors applied the Monte-Carlo least squares method to design microstrip resonators and their combination to design microstrip equalizers. This shows that there are many different approaches to design microwave devices; however, there are only a few, which rely on using differential evolution, e.g., ref. [18], where it was used for designing a sixth-order filter.

The focus of this experimental study is on the development of an approach, which would allow for discovering new types of microstrip resonator designs by means of evolutionary algorithms. The typical microstrip filter is usually in a form of a square or rectangular frame, or a closed meander line, but it is obvious that there are many other possibilities. The recent advances in the area of evolutionary computation [19], and especially differential evolution [20], allow us to formulate the problem of searching for new types of designs as a zero-order optimization task, which can be solved using state-of-the-art optimization techniques. In particular, in this study, we consider the L-SRTDE algorithm [21], which took the first place in the Congress on Evolutionary Computation 2024 competition on single-objective bound-constrained numerical optimization. The search space of possible designs in this study encoded by the positions of two rectangles, which are mirrored horizontally and vertically in order to create a symmetric structure. The performed numerical experiments results allow us to formulate the following important findings:

- The design of the target function has a huge influence on the final results; however, the L-SRTDE algorithm was able to successfully find suboptimal solutions in all experiments.
- Some of the constructions created during the experiments are different from any known resonator designs; however, most of them have commonalities with existing ones.

The rest of this paper is organized as follows. The next, Section 2 of this paper describes the topologies and properties of microstrip resonators, as well as differential evolution algorithm in general and the modification L-SRTDE used here. Section 2 also describes the proposed approach, in particular the encoding scheme of the search space, and shows several possible designs, as well as the two target function formulations used in the experiments. The Section 3 contains the experimental results, demonstrates some of the best found designs, and analyzes their characteristics, as well as the optimization algorithm convergence. Section 4 concludes the paper.

## 2. Materials and Methods

### 2.1. Related Work: Microstrip Resonators

The microstrip resonators considered in this study, which typically operate in microwave range, are frequency-selective devices, which are characterized by their amplitude-frequency characteristics (AFCs). The quality of the resonator is characterized by a certain functional criteria, calculated based on the AFC. Here the planar resonators are considered, which are manufactured on a monolithic substrates made of either TBNS (ceramic material based on a solid solution of barium, neodymium, and samarium titanates) or Policor with a thickness of h=1 mm and different relative permittivity ε=80 and ε=9.8 respectively. In this subsection, one of the widely used topologies of microstrip resonators is considered, namely the U-shaped one, whose bandwidth is created by its two or three lowest oscillation modes.

**Figure 1:**
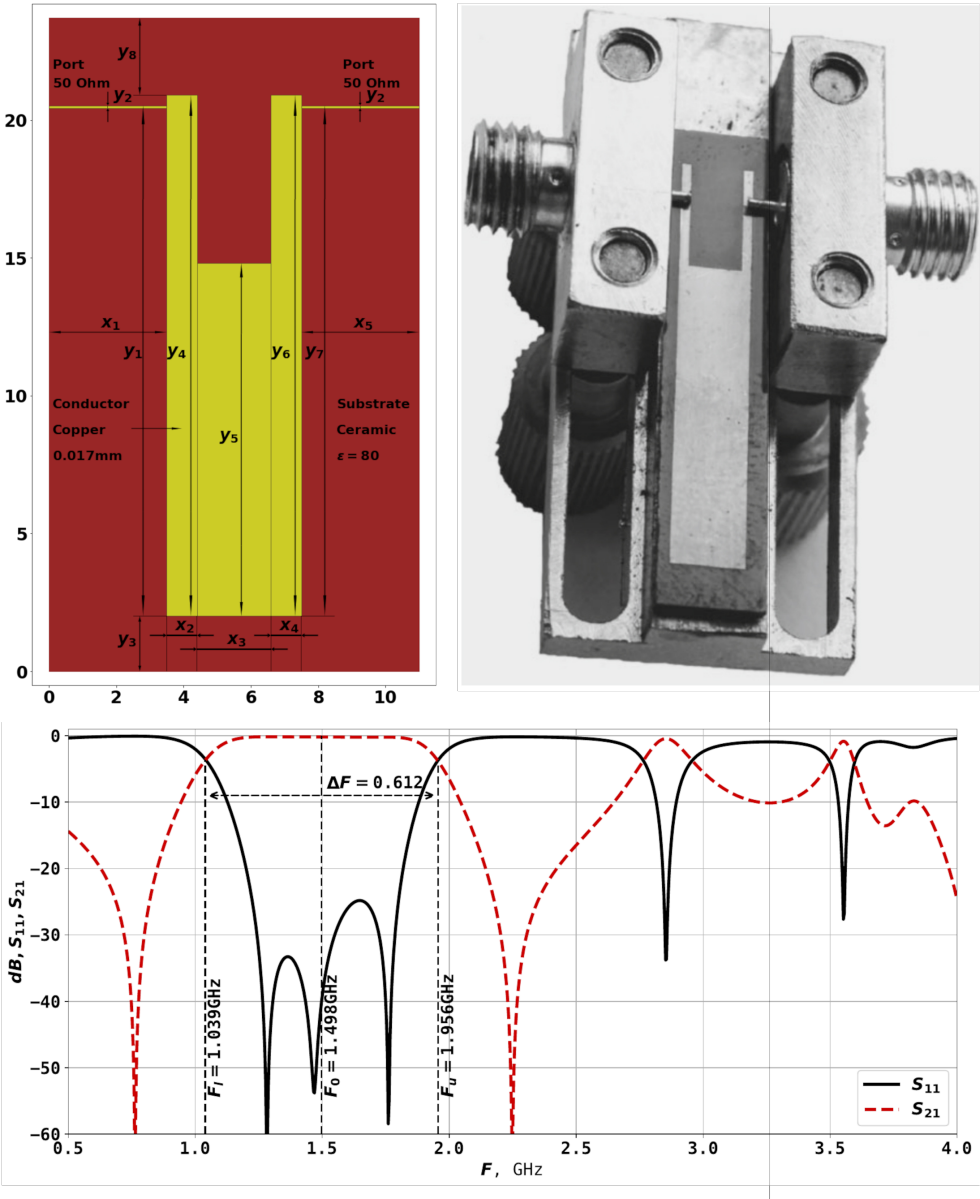
Topology of the U-shaped resonator, photo of a manufactured device of the same topology, and the amplitude-frequency characteristic of the resonator (S-parameters) [23].

Figure 1 shows an example of U-shaped resonator, manufactured example of the same topology, as well as the AFC, as was described in [22,23].

Changing the width and depth of the cut of the resonator in Figure 1 allows to bring together the lower frequencies of the resonator. The bandwidth is the frequency range within which the amplitude-frequency response of a radio device is uniform enough to ensure the transmission of a signal without significant distortion of its shape. The bandwidth is defined as the difference between the upper and lower bandwidth boundary frequencies. These frequencies are measured at a level of around −3 dB from the minimum bandwidth loss level. The size of the resonator itself influences the bandwidth range, and smaller sizes result in high frequencies. In the example here, the lower frequency boundary of the bandwidth $F_{l}$ is 1.039 GHz, the upper frequency boundary $F_{u}$ is 1.956 GHz, and the center frequency $F_{0}$ is 1.498 GHz. The bandwidth is calculated as $dF=\frac{F_{h}-F_{l}}{F_{0}}$ and equals 0.612 for the example in Figure 1. By changing the geometric characteristics of the resonator, such as x1–x5 and y1–y7, it is possible to obtain different AFCs for the given task.

There are many other types of resonator structures, for example U-shaped with an additional cut, or W-shaped, and the space of possibilities is enormous. We do not intend to cover all of them here, but rather give the basic description of the simplest typical resonator, as the aim of the study is to discover new topologies.

### 2.2. Related Work: Differential Evolution

As the main idea of the study is to search for new topologies, an appropriate optimization technique is required to perform this search. The search problem is formulated here as a black-box optimization problem, where given an evaluation function f(x), *x* being the parameter set, the best value of this function should be found, as well as the corresponding parameter set, i.e., $f(x^{*})\to opt$. The black-box formulation means that no information about the properties of the target function is given, and the gradient information is not available as well. So, the algorithm should rely purely on the target function values in order to proceed exploring the search space in pursuit of the best solution.

The described optimization problem can be solved by a zero-order algorithm; however, in recent years, the differential evolution algorithm [24] has become very popular due to its high efficiency, small number of parameters and easy implementation [25,26]. In the DE literature, many variants are described, and most of them focus on eliminating the main drawback of DE, i.e., its high sensitivity to parameter values [27].

Same as most evolutionary algorithms, differential evolution (DE) starts by initializing a set of NP solution vectors $x_{i}=\left\{x_{i,1},x_{i,2},\ldots ,x_{i,D}\right\}$, where *D* is the search space dimension. These vectors are initialized randomly within the search boundaries $\left[x_{min,j},x_{max,j}\right]$, where j=1,…,D. After initialization, the main loop of DE begins, which contains three main operations:

1. Mutation: $v_{i,j}=x_{r1,j}+F\left(x_{r2,j}-x_{r3,j}\right)$, where *F* is the scaling factor parameter, and $v_{i,j}$ is the mutant vector;
2. Crossover: $u_{i,j}=\left\{\begin{array}{cc} v_{i,j}, & \mathrm{if}\;rand(0,1)\leq Cr\;\mathrm{or}\;j=jrand\\ x_{i,j}, & \mathrm{otherwise} \end{array}\right.$, where Cr is the crossover rate parameter, and $u_{i,j}$ is the trial vector;
3. Selection: $x_{i,j}=\left\{\begin{array}{cc} u_{i,j}, & \mathrm{if}\;f\left(u_{i}\right)\leq f\left(x_{i}\right)\\ x_{i,j}, & \mathrm{if}\;f\left(u_{i}\right)>f\left(x_{i}\right) \end{array}\right.$, that is, if the trial vector is better than the target vector $x_{i,j}$, then replace it.

In the mutation step, the indices r1, r2, and r3 are generated randomly different from each other. The described mutation strategy is called rand/1 and is one of the first proposed. In the JADE algorithm [28], the current-to-pbest strategy was developed, which works as follows:

(1) $$v_{i,j}=x_{i,j}+F\left(x_{pbest,j}-x_{i,j}\right)+F\left(x_{r1,j}-x_{r2,j}\right).$$

where $x_{i}$ is the *i*-th vector in the population, pbest is the index of one of the p% best individuals, and r1, r2 are selected randomly. Current-to-pbest is one of the most widely applied strategies, since used in the L-SHADE algorithm [29] and its successors [25].

The L-SHADE algorithm has also proposed the tuning of the three main parameters of DE, the scaling factor *F*, crossover rate Cr, and population size NP. The *F* and Cr are tuned based on the success history, where values which resulted in better solutions are used to steer the adaptation, while the population size is linearly reduced. In addition, L-SHADE also has an external archive of solutions excluded from the population—these are used in mutation to increase the diversity.

Recently several studies have attempted to change the general scheme of DE; for example, in [30], the population was unlimited in size and different selection schemes were applied to steer the search process. Inspired by this work, in [26], a dual-population algorithm L-NTADE was proposed, where one population contained the best solutions, and the other—newest—solutions. Applying a hyper-heuristic approach to this idea led to the development of L-SRTDE algorithm [21], which won the CEC 2024 competition on numerical optimization [31] and was used further in this study.

The mutation strategy used in the L-SRTDE algorithm is called r-new-to-ptop/n/t and works as follows:

(2) $$v_{i,j}=x_{r1,j}^{new}+F\cdot \left(x_{pbest,j}^{top}-x_{i,j}^{new}\right)+F\cdot \left(x_{r2,j}^{new}-x_{r3,j}^{top}\right)$$

where r1 and r3 are chosen randomly, r2 is chosen with rank-based selective pressure with kp=3, and pbest is chosen from pb% best solutions in the top population. This strategy uses a randomly chosen individual as a basic one, instead of current. Another feature of L-SRTDE is the replacement scheme. Instead of comparing an individual with its previous version and replacing it, here another individual is replaced:

(3) $$x_{nc}=\left\{\begin{array}{cc} u_{i}, & \mathrm{if}\;f\left(u_{i}\right)\leq f\left(x_{r1}^{new}\right)\\ x_{nc}, & \mathrm{if}\;f\left(u_{i}\right)>f\left(x_{r1}^{new}\right) \end{array}\right..$$

where nc is an index, iterated after every successful replacement, and if nc>N, it is reset to 1. This leads to a continuous update of the population of the newest solutions. The second population with the top solutions is updated by collecting all individuals created within a generation, and then sorting them and taking only NP best ones.

L-SRTDE also uses a specific parameter adaptation technique, where the scaling factor *F* is set based on the success rate SR, i.e., relative number of times NS when selection worked:

(4) mF=0.4+0.25·tanh(5·SR)

where $SR=\frac{NS}{N}$. The same SR value is used to set the greediness parameter pb in mutation:

(5) $$pb=0.7\cdot e^{-7\cdot SR}.$$

In addition to this the size of both populations is linearly reduced during the search process as follows:

(6) $$NP_{g+1}=round\left(\frac{NP_{min}-NP_{max}}{NFE_{max}}NFE+NP_{max}\right),$$

where NFE is the current number of target function evaluations, $NFE_{max}$ is the total computational resource, and *g* is the generation number. That is, in the beginning, the populations have large number of vectors to cover as many regions of the search space as possible, and closer to the end, the population size reduces, allowing us to concentrate the search efforts on the most promising region.

### 2.3. Proposed Approach: Solutions Encoding Scheme

In order to efficiently represent a large variety of possible topologies, the encoding scheme should allow generating resonators which are diverse in their properties, but also have as few parameter settings as possible. To increase the variety of possible solutions, the topologies are designed with two symmetries, mirroring horizontally and vertically. The horizontal mirroring is applied always, while the vertical can be triggered by the optimization algorithm.

The resonator is represented by a pair of rectangles—their positions and sizes are the main parameters tuned by the optimization algorithm. The rectangles may overlap, creating various shapes.

Note that the overlapping areas are considered as having a single layer of conductor, not two, so the order of overlapping does not have any influence. In the case if a small rectangle is fully within the big one, it is equivalent to having only a single big rectangle.

The last part of the encoding is the port positioning scheme. Each port may have three possible positions, namely from the top, bottom, or side. Unlike the rectangles, the port positions are not mirrored, so it is possible that, for example, on the left side, the port is connected from the top, and on the right side—from the bottom.

**Figure 2:**
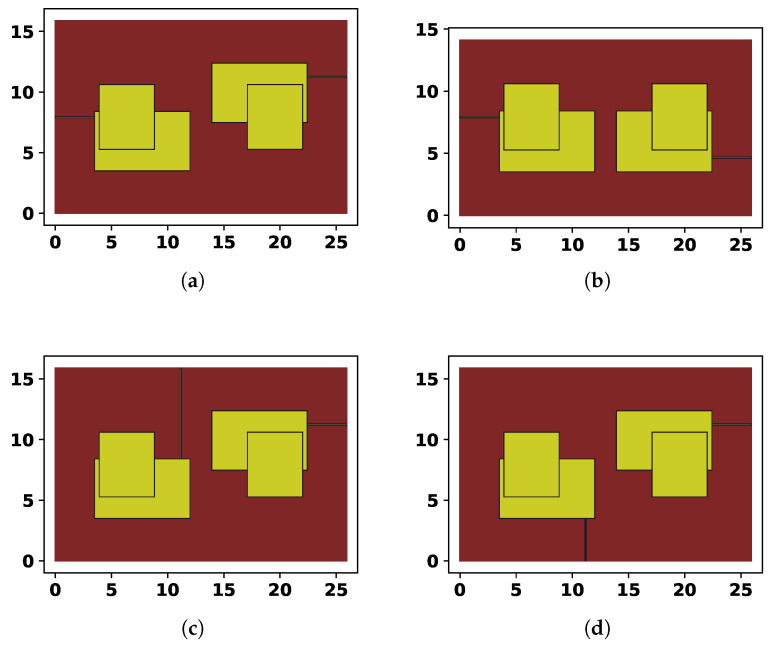
Four examples of generated topologies: (**a**) Randomly generated solution. (**b**) Same solution, but without vertical mirroring. (**c**) The position of the left port changed from side to top. (**d**) The position of the left port changed to bottom.

An example of a randomly generated solution, and its possible alternatives is shown in Figure 2. The 13 parameters of the topology encoding are the following:

1. $x_{1}\in \left[0,1\right]$—relative position of the port on the left side;
2. $x_{2}\in \left[0,1\right]$—relative position of the port on the right size;
3. $x_{3}\in \left[0.2,10\right]$—width of the first rectangle, in mm;
4. $x_{4}\in \left[0,10\right]$—vertical position of the first rectangle, in mm;
5. $x_{5}\in \left[0.2,10\right]$—height of the first rectangle, in mm;
6. $x_{6}\in \left[0,10\right]$—horizontal position of the second rectangle, in mm;
7. $x_{7}\in \left[0.2,10\right]$—width of the second rectangle, in mm;
8. $x_{8}\in \left[0,10\right]$—vertical position of the second rectangle, in mm;
9. $x_{9}\in \left[0.2,10\right]$—height of the second rectangle, in mm;
10. $x_{10}\in \left[-1,5\right]$—size of the gap between left and right parts of the resonator, in mm;
11. $x_{11}\in \left[0,1\right]$—indicates whether the vertical reflection should be applied;
12. $x_{12}\in \left[0,1\right]$—controls the left port positioning;
13. $x_{13}\in \left[0,1\right]$—controls the right port positioning.

The gap between the two mirrored sides of the resonator can be set to negative value, to allow overlapping. The vertical reflection is set to true of $x_{11}<0.5$, the position of the left port is set to side if $x_{12}\in \left[0,\frac{1}{3}\right]$, top if $x_{12}\in \left[\frac{1}{3},\frac{2}{3}\right]$ and bottom if $x_{12}\in \left[\frac{2}{3},1\right]$, same is true for $x_{13}$. The exact parameter values for the case in Figure 2 are shown in Table 1. The configurations in Figure 2 were considered to show how the encoding scheme works, in particular, how the same solution is altered by placing the ports on different sides and reflecting the left part of the topology to the right.

The distance from the edge of the substrate and the main rectangles is always set to 3.5 mm, regardless of the positioning and mirroring of the resonator. The size of the substrate is adjusted accordingly, as can be seen in Figure 2 by the height difference between cases (a) and (b). The length of the ports is also set to 3.5 mm where it is possible; for example, in case (c), the second rectangle is above the first one, so the port is longer than it should be. Such cases could be problematic for manufacturing, so they are penalized in the target function.

**Figure 3:**
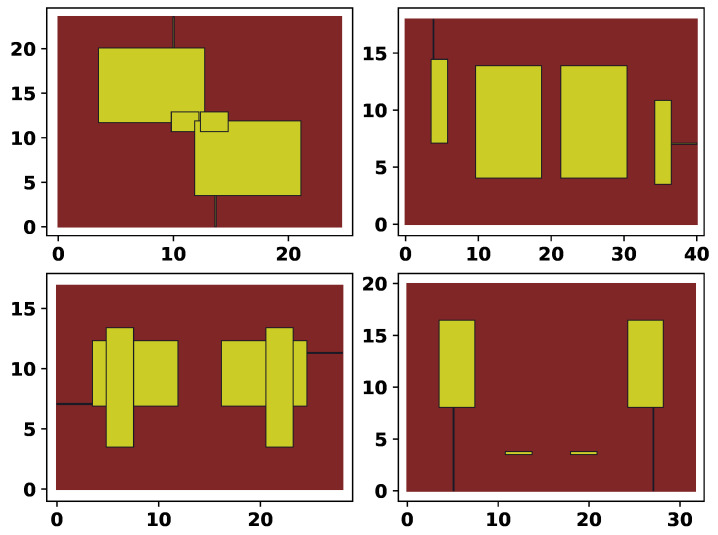
Four examples of random topologies.

In Table 1, the values of $x_{1}1$ and $x_{1}2$ are changed manually to demonstrate the effects of the mirroring and ports positioning. To show various examples generated by the described encoding scheme, Figure 3 shows four random topologies.

### 2.4. Proposed Approach: First Target Function Formulation

In the previous subsection, the solution encoding scheme was described in detail; however, in order to create an approach for the design of new resonator topologies, it should be coupled with a target function, which would direct the search process. The target function should represent the requirements to the properties of a microstrip resonator in a numeric form. Here, we propose two target function formulations, which will be further used in the experimental section.

The first target function takes into account the following parameters. The S-parameters of the resonator, evaluated through simulation, are analyzed to find the points where $S_{11}$ and $S_{12}$ intersect—these points mark the beginning and the end of the bandwidth (see Figure 1. They will be further referred to as $F_{l}^{act}$, $F_{u}^{act}$, i.e., lower actual frequency and upper actual frequency. In contrast, the desired lower and upper frequencies are given as $F_{l}^{des}$, $F_{u}^{des}$. The center frequency can be calculated as $F_{0}^{des}=0.5\cdot \left(F_{l}^{des}+F_{u}^{des}\right)$, same for actual frequencies. The relative bandwidth is therefore calculated as follows:

(7) $$dF^{des}=\frac{F_{u}^{des}-F_{l}^{des}}{F_{0}^{des}}.$$

The nominator in this equation is the bandwidth, i.e., $\Delta F^{des}=F_{u}^{des}-F_{l}^{des}$. Based on electrodynamic modeling, the same actual values are calculated.

In addition to this, the losses in reflection, i.e., the graph of the $S_{11}$ parameter should not exceed the loss threshold $LT^{des}=-14$ dB level. The −14 dB level is used as it is a typical setting for maximum acceptable losses within the bandwidth, according to authors’ contacts from manufacturing. This threshold can be set to any other desirable value, according to the requirements, as the proposed approach is flexible to this setting. The actual losses $LT^{act}$ should be greater than this value.

In order to bring all the lower bands of the resonator together, the next frequency point where the $S_{11}$ and $S_{12}$ intercross is found and is called $F_{n}^{act}$. This frequency should be brought down by the optimizer, so one of the subobjectives is minimize the index $I_{n}^{act}$ of the point where $F_{n}^{act}$ is met by dividing it by the number of points in the *S*-parameters evaluation, which was 1001 in the experiments.

The first target function in this study is different from the one used in [23]; in particular, the bandwidth is not taken into consideration. Instead, the excessive ports length is penalized. The first target function has the following five components, called fitness function components $fit_{1}$–$fit_{5}$:

1. Difference between the desired and actual center frequencies, $fit_{1}=\left|1-\frac{F_{0}^{act}}{F_{0}^{des}}\right|$.
2. Reflection losses being over the −14 dB threshold, $fit_{2}=\frac{LT^{act}}{LT^{des}}$ if $LT^{act}>LT^{des}$; otherwise, $fit_{2}=0$.
3. The actual number of nodes $NM^{act}$ should be as close as possible to the desired, $fit_{3}=\frac{NM^{des}-NM^{act}}{NM^{des}}$ if $NM^{act}<NM^{des}$; otherwise, 0.
4. The ports length $PL_{l}$ and $PL_{r}$ should be less then 1% longer than the gap between the edge of the substrate and the main rectangles, which is 3.5 mm. That is, $fit_{4}=max\left(PL_{l}/3.5\mathrm{if}PL_{l}>3.5\right),\left(PL_{r}/3.5\mathrm{if}PL_{r}>3.5\right)$.
5. The distance to the next band, if found, i.e., $fit_{5}=I_{n}^{act}/1001$, otherwise $fit_{5}=1$.

The resulting target function, which is also the fitness function (in evolutionary computation terminology) to be optimized by the differential evolution, is then formulated as follows:

(8) $$fit=fit_{1}+fit_{2}+fit_{3}+fit_{4}+fit_{5}.$$

**Figure 4:**
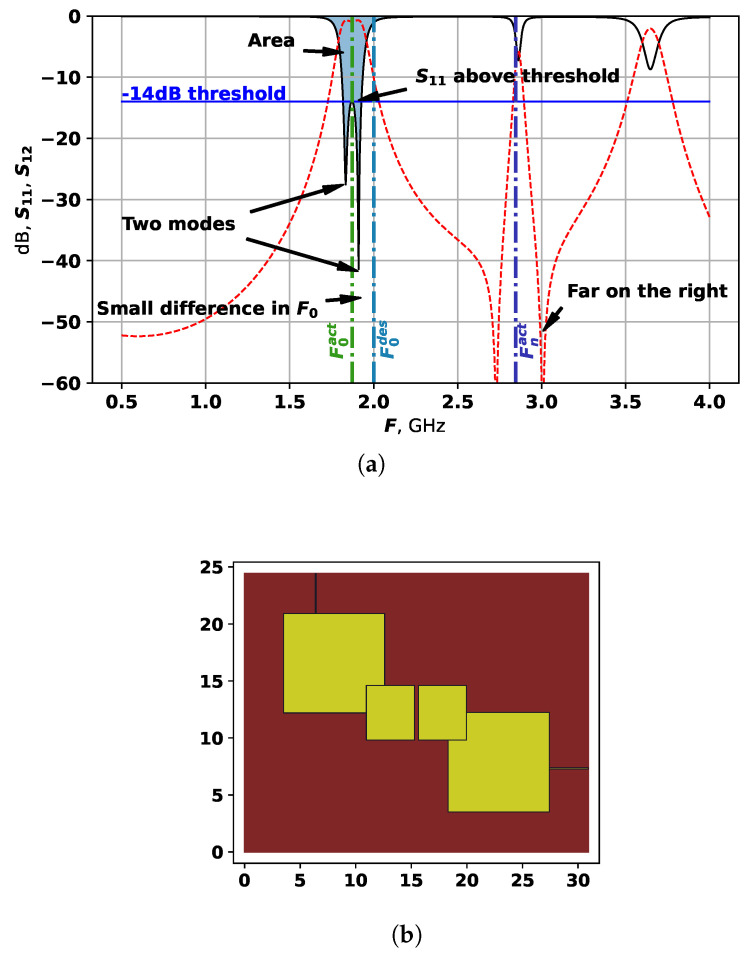
Example of target function calculation: (**a**) *S*-parameters analysis. (**b**) Topology.

To demonstrate how this target function works, Figure 4 shows each part of it on an example. The red line in Figure 4 is the $S_{21}$, and the black line is $S_{11}$, the blue line shows the −14 dB threshold.

For the example in Figure 4, the fitness values are the following:

1. $fit_{1}=0.064$;
2. $fit_{2}=0.006$;
3. $fit_{3}=-1$;
4. $fit_{4}=0$;
5. $fit_{5}=0.669$.

The value if $fit_{3}$ is equal to −1 as the desired number of modes is set to 1. Therefore, this particular solution is relatively good considering all parameters, however, can still be improved. The next mode frequency $F_{n}^{act}$ is quite far from the main two modes, but and if it can be moved to the left, it would increase the bandwidth.

### 2.5. Proposed Approach: Second Target Function Formulation

The second target function relies on calculating the area, taken by the bandwidth, and also aims at distributing the modes on equal distances from each other. The area is estimated by evaluating the sum of all $S_{11}$ values between $F_{l}^{act}$ and $F_{u}^{act}$. In other words, the first subobjective here is calculated as follows:

(9) $$fit_{1}=\sum_{i=I_{l}^{act}}^{I_{u}^{act}}S_{11,i}.$$

As $fit_{1}$ is a negative value, and the optimization method is set to solve minimization problems, by minimizing $fit_{1}$, we search for the largest area. The selected area is shown in Figure 4a.

The second part of the target function, i.e., based on reflection losses, is calculated in the same way as before, i.e., $fit_{2}=\frac{LT^{act}}{LT^{des}}$ if $LT^{act}>LT^{des}$, otherwise $fit_{2}=0$.

The last part of the target function is responsible for the even distribution of the modes. Based on the number of observed modes $NM^{act}$, their normalized ideal positions are calculated, that is $IM_{j}=\frac{j+1}{NM^{act}-1}$. We only care about the modes which are in the middle, that is if there are three modes, then the only ID value would be 0.5. In case of 4 modes, the values would be 1/3 and 2/3 respectively.

Based on the ideal positions of the modes, the difference between them and the normalized actual positions is calculated. The normalized actual positions are calculated as follows:

(10) $$AM_{i}=\frac{m_{i}^{act}-m_{0}^{act}}{m_{NM^{act}}^{act}-m_{0}^{act}}$$

where $m_{i}$ is the position of the *i*-th mode, and $I_{m_{i}}$ is its index. Next, the modes starting from the second one are compared to their ideal positions, and the third fitness subfunction is calculated:

(11) $$fit_{3}=\sum_{i=2}^{NM^{act}-1}\left(IM_{i-1}-AM_{i}\right).$$

If there are less then two modes, then $fit_{3}$ is always zero.

Finally, the second target function is calculated as follows:

(12) $$fit=\frac{fit_{1}\cdot \left(NM^{act}-fit_{3}\right)}{{\left(1+fit_{2}\right)}^{4}}.$$

This particular form of the target function has been designed based on the results of the first set of experiments with the first target function. That is, all the generated constructions were re-evaluated based on the previously calculated *S*-parameters, the target function was tuned so that the solutions which are as close as possible to the desired form, are ranked higher than any other. This analysis lead to the conclusion, that the area should be divided by the function depending on $fit_{2}$, which is greater than zero only if the −14 dB threshold is violated. So, if $LT^{act}>LT^{des}$, then this violation is added to 1 and raised to the power 4.

## 3. Results and Discussion

As the described approach relies on the direct evaluation of the generated topologies with electrodynamic modeling, it requires a huge computational effort to run the algorithm. The evaluation of a single topology took from 30 to 300 s, depending on the particular topology. So, to speed up the calculations, the evaluation of solutions was parallelized into eight computers, with eight cores each. The L-SRTDE algorithm, which was originally implemented in C++ 11, was re-written in Python 3.10, and both target functions were also implemented in Python 3.10. The following parameters were used during modeling: the tangent delta el = 0.001 at frequency 1 GHz, metal thickness is 0.01778 mm, relative permittivity of dielectric was set to ε=80, which corresponds to ceramic material based on a solid solution of barium, neodymium and samarium titanates. The frequency domain solver was used for calculating the S-parameters. We used frequency domain solver with broadband sweep, and adaptive tetrahedral mesh refinement, frequency range from 0.5 to 4.0 GHz.

The two computational experiments were performed, and only parameter changed for the L-SRTDE algorithm was the population size, set to NP=20·D, where D=13 is the problem dimension; all other parameters were the same as for the original L-SRTDE [21]. The computational resource for both experiments was set to 50,000 evaluations; however, the first experiment was stopped after 34,006 evaluations, as it was observed that the algorithm has converged. The first experiment required around four weeks to complete, while the second took only two weeks. The time difference is due to the fact that two target functions generated different solutions, and the second target function generated solutions which are faster to evaluate.

### 3.1. Results of the First Experiment

**Figure 5:**
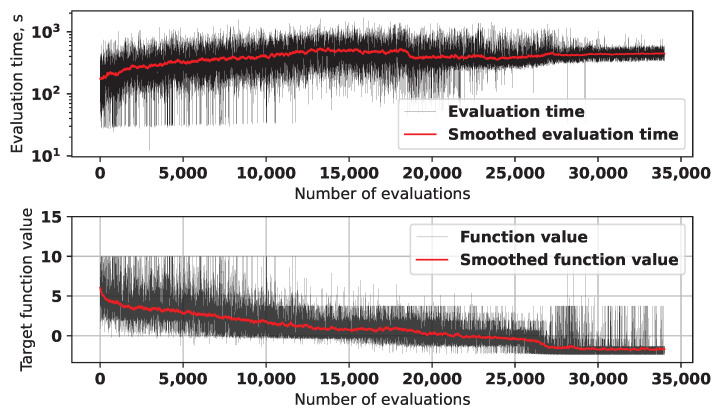
Evaluation time and target function values during the first experiment.

Figure 5 shows the graphs for the target function values and time required to evaluate solutions during the first run of the algorithm.

The red lines in both graph represent the values, obtained with exponential smoothing. Some of the solutions in the beginning of the optimization process created errors during the electromagnetic modeling, so their evaluation was aborted and fitness set to a high value (50). As can be seen from the graph, after around 25,000 evaluations the L-SRTDE algorithm has found a solution with high quality, and the next 9000 evaluations there were little progress. This was the reason why the optimization process was stopped, apart from the fact that it took a very long time.

**Figure 6:**
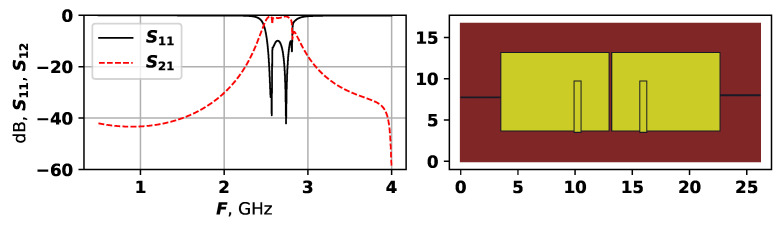
The AFC and topology of the best found solution during first experiment.

Figure 6 shows the best solution found by the proposed approach in the first experiment.

**Figure 7:**
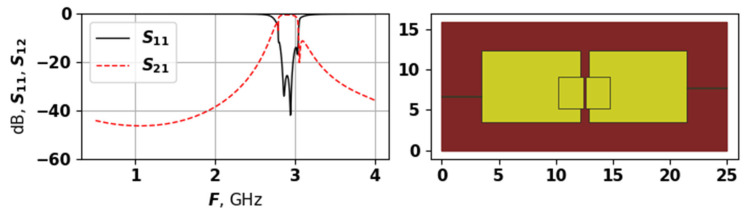
The AFC and topology of solution 9704, first experiment.

**Figure 8:**
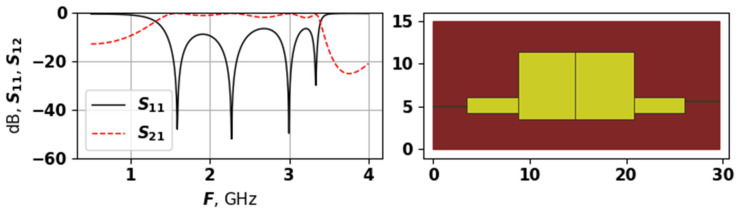
The AFC and topology of solution 9703, first experiment.

**Figure 9:**
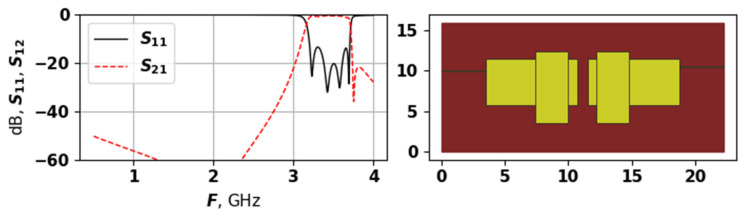
The AFC and topology of solution 4856, first experiment.

As Figure 6 shows, the best solution has a relatively simple structure, with two large rectangles, and a small patches on their lower parts. The ports connections are almost symmetrical. Nevertheless, this solution has five modes, and even though most of them are suppressed, it has the lowest target function value of −2.384. The solutions received after 25,000 evaluations are very similar to this one; however, earlier solutions are quite different. Figure 7, Figure 8 and Figure 9 show examples of generated topologies.

As Figure 7, Figure 8 and Figure 9 demonstrate, the generated topologies are quite different from each other. The one in Figure 8 is violating the −14 dB level but has a wide bandwidth, and a four modes, which are positioned with relatively equal distances. Solutions from Figure 7 and Figure 9 are similar in their AFC, but have very different topology, the one in Figure 9 does not violate the −14 dB level and has evenly spread modes, while the one from Figure 7 is closer to the target frequency range but has suppressed modes. These graphs demonstrate that the proposed approach is capable of generating a variety of unique topologies with different properties.

### 3.2. Results of the Second Experiment

Based on the results of the first experiment, an alternative method of evaluating solutions was proposed. In particular, as mentioned before, the results of the first experiment were ranked according to the new target function so that it was tuned to get the most promising types of topologies.

**Figure 10:**
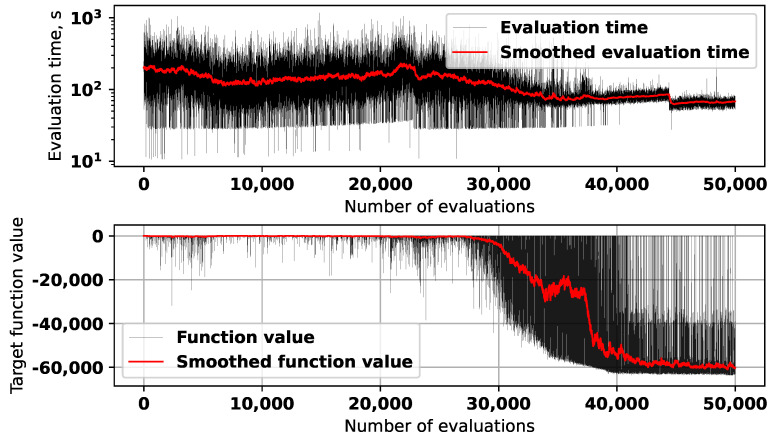
Evaluation time and target function values during the second experiment.

The second experiment ran for 50,000 target function evaluations, and in Figure 10, the convergence graph and the evaluation time are shown.

**Figure 11:**
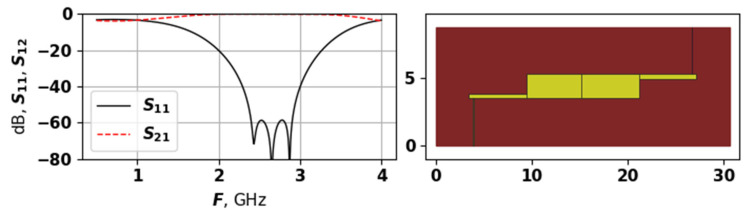
The AFC and topology of the best found solution during second experiment.

As the upper part of Figure 10 demonstrates, the average evaluation time during the experiment with the second target function stayed around 100 s. As for the convergence graph, for the first 25,000 evaluations, the algorithm was in the exploration phase, discovering some promising solutions occasionally. After this, one of the promising regions was identified, and the fast convergence process was started. By the moment when 40,000 were reached, the algorithm basically converged to the best solution with number 41,261, which is shown in Figure 11.

The solution shown in Figure 11 has a very wide bandwidth, and also the peaks of reflection losses between modes are at the level of −60 dB. The topology is very symmetric and regular, and also quite compact. The best solution has only 3 modes, but they are placed at an ideal distance from each other, that is, the value of the third function $fit_{3}$ equals zero. However, the slopes of the $S_{11}$ are very long and smooth, which may be not a good thing for certain applications—for example, where a narrow bandwidth is required. Nevertheless, this solution actually achieves all three goals, which were included in the target function.

**Figure 12:**
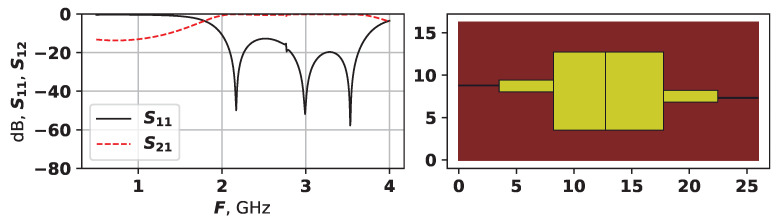
The AFC and topology of solution 23855, second experiment.

**Figure 13:**
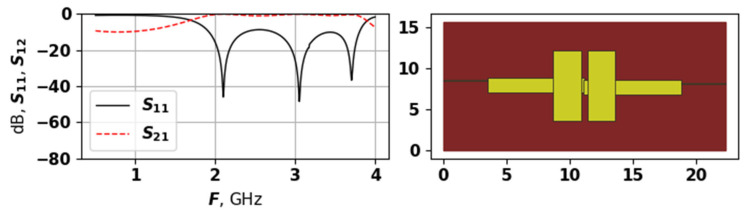
The AFC and topology of solution 2351, second experiment.

**Figure 14:**
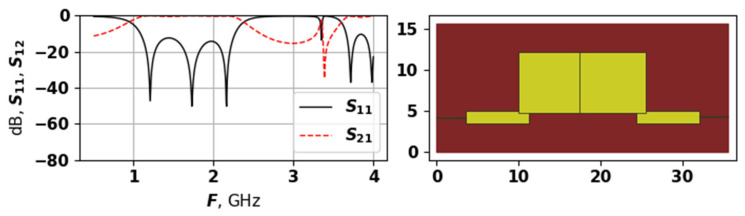
The AFC and topology of solution 22467, second experiment.

Three other interesting solutions obtained during this experiment are shown in Figure 12, Figure 13 and Figure 14.

The solution in Figure 12 has four modes, but two of them are very small, and are located at around 2.7 GHz. The solution in Figure 13 has four modes, and again, one of them is not really seen. This solution also has an odd symmetry, with overlapping horizontal rectangles between the two vertical ones. The solution in Figure 14 is similar to a more classical one, e.g., compare it to the hand-designed in Figure 1—it also has quite regular AFC. Solutions in Figure 12 and Figure 13 were not ranked very high, as the did not meet the requirement for the even distribution of modes. Such cases are the main reason why $fit_{3}$ was added into the target function for the second set of experiments—without it the search algorithm created a large number of barely seen suppressed modes, as it allowed achieving high target function values.

The experimental results shown here demonstrate that the combination of the L-SRTDE algorithm and the specific target function allowed generating a variety of microstrip resonator topologies, and the most promising of them can be further explored manually.

### 3.3. Analysis of Algorithm Convergence and Diversity

**Figure 15:**
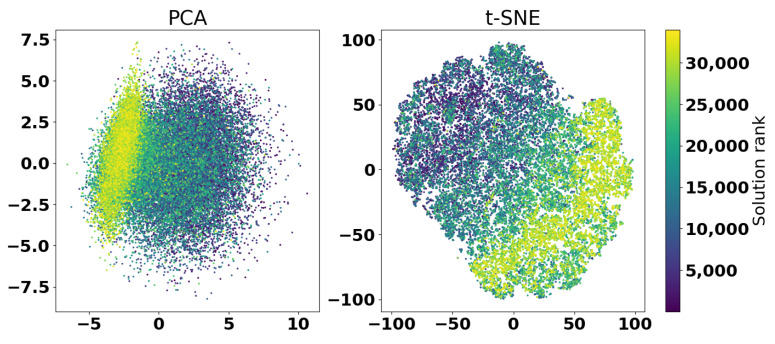
Visualization of solutions diversity and quality of the first experiment with PCA and t-SNE.

In order to analyze how the L-SRTDE converged and which solutions it explored in the search space, the coordinates of all tested solutions in both experiments were scaled down using Principal Components Analysis (PCA) [32] and t-SNE [33] approaches, with perplexity parameter set to 30. Figure 15 shows the results of transforming the coordinates of all solutions into the two-dimensional space for the first experiment.

The solutions in Figure 15 are ranked and colored according to their target function values. The ranks are used instead of fitness values directly to add contrast to the figure. As can be seen on the left side, the PCA shows that in the beginning the solutions are evenly distributed, but closer to the end, the best solutions are concentrated in a elliptic-shaped area. On the other hand, the t-SNE shows that there is a significant diversity among the best solutions—points with similar color are located far away from each other. This means that even 30,000 evaluations the L-SRTDE has a significant diversity in the population, which would allow continuing the optimization.

**Figure 16:**
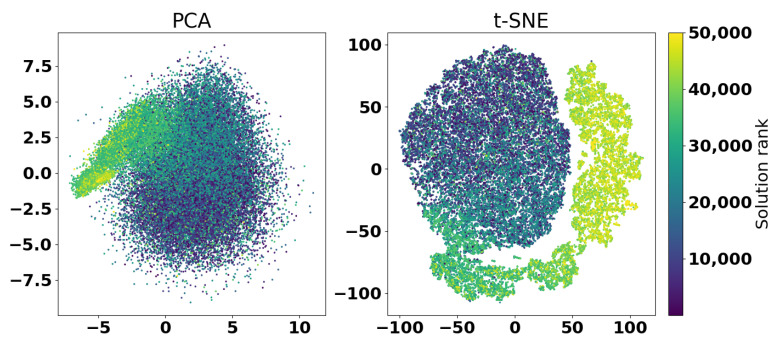
Visualization of solutions diversity and quality of the second experiment with PCA and t-SNE.

Figure 16 shows the PCA and t-SNE transformations of the results of the second experiment.

In Figure 16, it can be clearly seen how the optimization algorithm started from a relatively round set of solutions, but then continued concentrating on a particular area of the search space. This is especially seen in the t-SNE graph, where the movement of the points towards better solutions can be observed with several concentrated groups, which are further expanded as the search progresses. Considering the distribution of points, clustering methods could be applied to analyze the distribution of points to determine the convergence of the algorithm or increase diversity [34,35].

**Figure 17:**
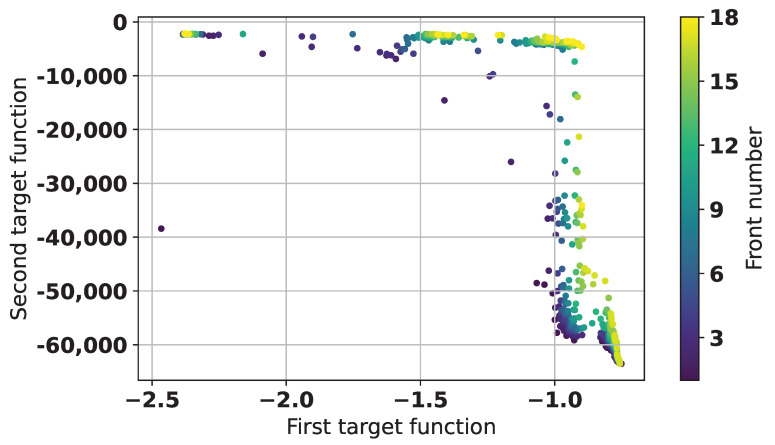
Results of non-dominated sorting of the data from two experiments, best 1000 solutions are shown.

To analyze the differences between two used objective functions and their effects on the found solutions properties, all of the obtained structures (34,006 + 50,000 totally) were additionally cross-evaluated by both objectives. Figure 17 shows the results of the non-dominated sorting of all solutions, in particular, only the best 1000 solutions are shown. There are 18 fronts in Figure 17, but the total number of fronts is 16,595.

**Figure 18:**
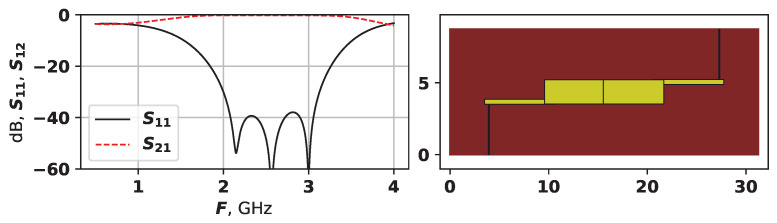
The AFC and topology of solution 38795, second experiment.

As can be seen from Figure 17, the two target functions used in the experiments are in fact different in their preferences, as they appear to form a structured picture of fronts. There is one solution on the left hand side, which is much better, than many others, as it is very good according to both objectives. This solution has number 57,861 in the joined set, which corresponds to 23,855 in the results of the second experiment—its S-parameters and topology has already been shown in Figure 12. Other members of the first Pareto front, which has 28 solutions, have similar structure to Figure 11, for example, solution with number 41,261, which has already been shown in Figure 14. There are also some trade-off solutions; however, they are also similar to solution 41,261. One of such is shown in Figure 18.

As can be seen from Figure 18, this solution has the same structure and very close parameter values, as it is taken from the period when the L-SRTDE algorithm has almost converged.

### 3.4. Discussion

The results of the two experiments shown in this paper demonstrate that given an appropriate target function, and a sufficient amount of computational resources, modern black-box optimization techniques, such as differential evolution, can be used to directly explore the space of microwave devices. Here the case of microstrip resonators was considered, but there are no hard limits to regarding the application area—if a physical system can be properly modeled with modern software, then it can not only be tuned, but also new types of solutions can be found.

The particular types of topologies found in this study may not be ready for manufacturing and application in the real world, as they require fine-tuning. However, the fine-tuning part has been thoroughly studied in many previous works, where hand-designed solutions are optimized with black-box algorithms. Moreover, the proposed approach is not limited to apply L-SRTDE algorithm—any other state-of-the-art optimizer can be used for such applications.

A limitation of this study is that it only focused on a single-objective case, whereas the design of real-world systems is almost always a multi-objective case. Here the target functions were carefully designed to combine five (in the first case) or three (in the second case) separate objectives in a single target function so that the optimization would yield satisfying results. The advantage of the single-objective approach is that it allows concentrating the search effort in a particular direction, while the multi-objective approach may have problems with convergence, especially if there are more than three objectives. Nevertheless, the application of the most advanced multi-objective algorithms to the same problem of multimode resonator design is an important direction of further research. As the analysis of the results of both experiments has shown, there is a significant gap in the middle of the obtained set of Pareto fronts, which were received after non-dominated sorting, so the application of a multiobjective algorithm which would combine the two target functions proposed in this study may allow finding topologies with even better characteristics.

## 4. Conclusions

This paper concentrated on an application of the modern evolutionary computation method, in particular the L-SRTDE algorithm, to the design of microwave devices, i.e., microstrip resonators. The direct search for the most promising solutions with two proposed target functions, possible due to the proposed encoding scheme, has allowed generating a variety of different solutions to the same problem, and the analysis of best results has shown that the topologies differ in shape and properties.

The described approach is not limited to resonators of this particular structure—the same method can be applied to other classes of devices, e.g., diplexers, power dividers, filters, antennas or sensors of different kinds. The analysis of the algorithm run has shown that the chosen optimization algorithm was capable of solving the problems successfully; however, it is hard to say how efficient it was, and if the results can be improved. The required number of evaluations of around 25,000 to start the quick convergence can be hard to achieve in some applications, as the evaluation time may be bigger, than in the current study. Hence, one of the directions of further research could directed toward determining the properties of such problems, and which methods could be efficiently used to solve them.

## Figures and Tables

**Table 1:** Parameter values for the cases in Figure 2.

	Case (a)	Case (b)	Case (c)	Case (d)
1	0.909	0.909	0.909	0.909
2	0.226	0.226	0.226	0.226
3	8.486	8.486	8.486	8.486
4	3.480	3.480	3.480	3.480
5	4.902	4.902	4.902	4.902
6	0.407	0.407	0.407	0.407
7	4.912	4.912	4.912	4.912
8	5.249	5.249	5.249	5.249
9	5.338	5.338	5.338	5.338
10	1.937	1.937	1.937	1.937
11	0.200	0.700	0.464	0.464
12	0.127	0.127	0.500	0.700
13	0.182	0.182	0.182	0.182

## Data Availability

The original contributions presented in this study are included in the article. Further inquiries can be directed to the corresponding author.

## Abbreviations

The following abbreviations are used in this manuscript:

AFC	amplitude-frequency characteristic
HPF	high-pass filter
EA	Evolutionary Algorithm
GA	Genetic Algorithm
L-SRTDE	Linear population size reduction Success RaTe-based Differential Evolution
NFE	Number of function evaluations (simulations)
PCA	Principal Components Analysis
t-SNE	t-distributed Stochastic Neighbor Embedding
